# Soil and landscape factors influence geospatial variation in maize grain zinc concentration in Malawi

**DOI:** 10.1038/s41598-022-12014-w

**Published:** 2022-05-14

**Authors:** L. Botoman, C. Chagumaira, A. W. Mossa, T. Amede, E. L. Ander, E. H. Bailey, J. G. Chimungu, S. Gameda, D. Gashu, S. M. Haefele, E. J. M. Joy, D. B. Kumssa, I. S. Ligowe, S. P. McGrath, A. E. Milne, M. Munthali, E. Towett, M. G. Walsh, L. Wilson, S. D. Young, M. R. Broadley, R. M. Lark, P. C. Nalivata

**Affiliations:** 1 Lilongwe University of Agriculture and Natural Resources (LUANAR), Bunda College Campus, P.O. Box 219, Lilongwe, Malawi; 2 The Department of Agricultural Research Services, P.O. Box 30779, Lilongwe 3, Malawi; 3 School of Biosciences, University of Nottingham, Sutton Bonington Campus, Nottinghamshire, LE12 5RD UK; 4 Future Food Beacon, University of Nottingham, Sutton Bonington Campus, Nottinghamshire, LE12 5RD UK; 5 Rothamsted Research, Harpenden, Hertfordshire, AL5 2JQ UK; 6 Alliance for Green Revolution in Africa (AGRA), o/C, ILRI, Guidoshola, P.O. Box 5689, Addis Ababa, Ethiopia; 7 Centre for Environmental Geochemistry, British Geological Survey, Keyworth, Nottinghamshire, NG12 5GG UK; 8 International Maize and Wheat Improvement Center (CIMMYT), ILRI Sholla Campus, P.O. Box 5689, Addis Ababa, Ethiopia; 9 Centre for Food Science and Nutrition, Addis Ababa University, P.O. Box 1176, Addis Ababa, Ethiopia; 10 Faculty of Epidemiology and Population Health, London School of Hygiene & Tropical Medicine, Keppel Street, London, WC1E 7HT UK; 11 World Agroforestry (ICRAF), United Nations Avenue, P.O. Box 30677, Nairobi, Kenya; 12 Africa Soil Information Service, Selian Agricultural Research Institute, P.O. Box 2704, Arusha, Tanzania

**Keywords:** Biogeochemistry, Environmental sciences

## Abstract

Dietary zinc (Zn) deficiency is widespread globally, and in particular among people in sub-Saharan Africa (SSA). In Malawi, dietary sources of Zn are dominated by maize and spatially dependent variation in grain Zn concentration, which will affect dietary Zn intake, has been reported at distances of up to ~ 100 km. The aim of this study was to identify potential soil properties and environmental covariates which might explain this longer-range spatial variation in maize grain Zn concentration. Data for maize grain Zn concentrations, soil properties, and environmental covariates were obtained from a spatially representative survey in Malawi (n = 1600 locations). Labile and non-labile soil Zn forms were determined using isotopic dilution methods, alongside conventional agronomic soil analyses. Soil properties and environmental covariates as potential predictors of the concentration of Zn in maize grain were tested using a priori expert rankings and false discovery rate (FDR) controls within the linear mixed model (LMM) framework that informed the original survey design. Mean and median grain Zn concentrations were 21.8 and 21.5 mg kg^−1^, respectively (standard deviation 4.5; range 10.0–48.1). A LMM for grain Zn concentration was constructed for which the independent variables: soil pH_(water)_, isotopically exchangeable Zn (Zn_*E*_), and diethylenetriaminepentaacetic acid (DTPA) extractable Zn (Zn_DTPA_) had predictive value (*p* < 0.01 in all cases, with FDR controlled at < 0.05). Downscaled mean annual temperature also explained a proportion of the spatial variation in grain Zn concentration. Evidence for spatially dependent variation in maize grain Zn concentrations in Malawi is robust within the LMM framework used in this study, at distances of up to ~ 100 km. Spatial predictions from this LMM provide a basis for further investigation of variations in the contribution of staple foods to Zn nutrition, and where interventions to increase dietary Zn intake (e.g. biofortification) might be most effective. Other soil and landscape factors influencing spatially dependent variation in maize grain Zn concentration, along with factors operating over shorter distances such as choice of crop variety and agronomic practices, require further exploration beyond the scope of the design of this survey.

## Introduction

Zinc (Zn) is an essential micronutrient with critical roles in human health^1^. Zinc deficiency among human populations is widespread^2,3^. In sub-Saharan Africa (SSA), Zn deficiency is likely to exceed 40% of the total population in many countries based on dietary Zn supply^3^. In Malawi, an estimated Zn deficiency prevalence rate of 62% was reported^4^, based on a national survey of serum Zn concentration conducted in 2015/16; this is likely to be higher in rural areas^5^. In Ethiopia, 72% of the population was reported to be Zn deficient^6^, also based on a national survey of serum Zn concentrations in 2015/16, also with a greater prevalence of deficiency in rural areas.

Many people in SSA rely on locally produced cereals for most of their dietary Zn supply and have limited access to Zn-rich plant and animal source foods^3,7^. The grains of most cereals are a poor source of dietary Zn, which is compounded by high contents of anti-nutritional compounds such as phytates (inositol phosphate compounds) which inhibit the absorption of Zn and other micronutrients in the human gut^7^.

Recent evidence has emerged of geospatial variation in grain Zn concentration in staple cereal crops in SSA, linked to soil properties and environmental covariates. This variation is likely to be of dietary significance in the context of dietary Zn supply, especially for rural populations. For example, in Malawi, the grain Zn concentration of maize grown on Vertisols was ~ 30% larger than the grain concentration from other soils^8^. Among smallholder communities farming on these Vertisols, a 35% greater dietary Zn supply was seen compared to other soils in Malawi, based on the analyses of composite diets^5^. Geospatial variation in Zn concentrations of staple crop grains has also been observed in Uganda, where smaller grain Zn concentrations were observed in sandier soils^9^. Evidence that soil properties and environmental covariates influence geospatial variation in the Zn concentration of maize grain has recently been reported in both Ethiopia and Malawi^10^. For example, soil pH and soil organic carbon (SOC) were predictive for maize grain Zn concentration (positive relationships) in both countries. Mean annual temperature and a topographic index—which indicates the potential water content of soils arising from drainage—were positively related to grain Zn concentration for maize in Malawi. The value of the variogram increased at distances up to 100 km in Malawi, providing robust evidence that soil properties and landscape factors can influence maize grain Zn concentration at multiple scales^10^.

The aim of this study was to gain a greater understanding of soil properties and environmental covariates influencing grain Zn concentration. The focus was on maize grain and co-located soils, sampled at locations that provide a wide spatial coverage of the cultivated soils of Malawi^10^. In addition to general soil properties such as soil pH and SOC, this study sought to characterise Zn in soils in greater detail. Thus, ‘geochemically-reactive’ (labile) fractions of Zn were measured using stable isotopes of Zn, alongside a broader suite of standard soil properties including ‘total’ (aqua regia) and diethylenetriaminepentaacetic acid (DTPA)-extractable Zn. Indices which characterise labile Zn in soils include the solution ⇌ solid phase distribution coefficient of an enriched isotopic spike (Zn_*K*d_), which indicates Zn mobility in soil, and the isotopically exchangeable Zn fraction (Zn_*E*_), which is considered to be a more accurate estimate of the potentially accessible pool to plants^11–13^. In a recent study in Amhara Region of Ethiopia, a positive relationship between Zn_*E*_ and soil pH was reported^13^, however, it is not yet known how this relationship might influence grain Zn concentration. For landscape factors, downscaled precipitation and temperature, terrain index (TIM), slope, and vegetation index were considered. An explicit, hypothesis testing approach was adopted, using a method to maximise power to detect significant predictors, based on expert ranking of the soil and environmental factors considered most likely, a priori, to influence grain Zn concentration and using false discovery rate (FDR) control. A similar approach was used previously to identify factors influencing grain selenium (Se) concentration in Amhara Region, Ethiopia^14^. More information on the procedure is provided in the “Materials and Methods” section.

## Results

### Exploratory analyses

The summary statistics for maize grain Zn concentration, together with residuals from model fitting and cross-validation errors (see Methods) are shown in Table 1.

**Table 1:** Summary statistics of Zn concentration in grain (n = 1600), of residuals from fitted exploratory saturated models and cross-validation errors for the E-BLUP with easting and downscales mean annual temperature as fixed effects.

	Concentration of Zn in maize grain mg kg^−1^	Residuals from model, soil properties as covariates	Residuals from model, environmental covariates	Cross-validation errors
Mean	21.8	0.00	0.00	0.00
Median	21.5	− 0.31	− 0.24	− 0.28
Minimum	10.0	− 12.19	− 11.84	− 10.91
Maximum	48.1	27.51	25.34	25.86
Standard deviation	4.5	4.21	4.11	3.98
Skewness	0.6	0.78	0.61	0.68
Octile skewness	0.05	0.07	0.07	0.09

The summary statistics for soil properties (Table 2) indicated that most of these were markedly skewed, except for Zn_*K*d_ and pH, which are both reported on logarithmic scales. Skewness coefficients for the other variables ranged from − 0.26 to 18.39, and the octile skewness ranged from − 0.10 to 9.52. All these variables were transformed to their natural logarithms prior to further analysis, as a result of which the conventional skewness coefficient and octile skewness were all restricted to the intervals [− 1,1] and [− 0.2,0.2], respectively. The summary statistics and exploratory plots for the residuals from an exploratory fit of the saturated models (all soil properties or all environmental covariates as fixed effects) indicated that an assumption of normality was plausible without any transformation of the data on grain Zn concentration (Supplementary Figures S1 and S2).

**Table 2:** Summary statistics of soil properties (n = 1600) proposed as predictors of Zn concentration in grain.

Variable	Original variables						Log_e_-transformed					Transformed
	Units	Mean	Median	Standard deviation	Skew	Octile skew	Mean	Median	Standard deviation	Skew	Octile skew	
Zn_AR*_	(mg kg^−1^)	39.60	32.9	38.79	12.11	0.27	3.45	3.49	0.66	0.02	− 0.08	Yes
Zn_S_	(mg kg^−1^)	0.22	0.05	1.39	18.39	0.74	− 2.78	− 2.95	− 1.29	0.53	0.19	Yes
Zn_DTPA_	(mg kg^−1^)	2.12	0.86	5.29	9.52	9.52	− 0.03	− 0.15	1.09	0.68	0.13	Yes
Zn_*E*_	(mg kg^−1^)	7.13	4.06	11.06	7.36	0.56	1.47	1.40	0.94	0.17	0.10	Yes
Zn_*K*d_	Log(L kg^−1^)	2.57	2.64	0.77	− 0.26	− 0.10						No
pH		6.32	6.25	0.66	0.66	0.13						No
SOC	(%)	1.11	0.95	0.64	1.76	0.31	− 0.04	− 0.05	0.52	0.17	0.02	Yes
Oxalates	(mg kg^−1^)	3683	3186	2339	2.78	0.24	8.06	8.07	0.54	0.23	− 0.06	Yes
eCEC	cmol_c_ kg^−1^	7.26	5.67	5.80	2.11	0.35	1.69	1.74	0.81	–0.84	− 0.07	Yes

*The subscripts AR, S, DTPA, *K*d and *E* denote the total (aqua regia extractable), soluble (calcium nitrate extractable), potentially available (DTPA extractable), the solid-solution distribution coefficient, and the isotopically exchangeable (isotopic dilution) fractions. SOC denotes soil organic carbon. eCEC denotes the effective cation exchange capacity. Oxalates denotes the sum of oxalate-extractable Fe, Al and Mn oxides.

### Ranking of predictor variables as predictors of Zn concentration in grain

As described in the “Materials and Methods” section, the identification of variables to include as fixed effects (covariates) in statistical models for the target variable of interest, was done by a sequential set of hypothesis tests about the candidate variables in turn, with control of the False Discovery Rate (FDR) to allow for the fact that this process involves testing of multiple hypotheses. To maintain the power with which valid predictors are detected, a method called α-investment was used. This method is most effective when the tests are conducted in a sequence with the predictors most likely to be related to the target variable considered first. Before examination of the data a ranking of the soil properties and of the environmental covariates was elicited from an expert panel, and the rankings are presented in Table 3, with the first-ranked property the one thought most likely to be predictive of Zn concentration in grain. The first-ranked soil property, Zn extracted in calcium nitrate, is interpreted as a measure of soluble Zn and is an intensity variable, related to the concentration of Zn in the soil solution. Such Zn could be expected to be readily available to the growing crop. The next variable, pH, was included because of its potential effects on the uptake of Zn by the plant.

**Table 3:** Sequence of predictors for grain Zn concentration (both soil properties and environmental covariates) for testing with the α-investment.

Order	Soil property	Order	Environmental covariate
1	Zn_S*_	1	Downscaled mean annual precipitation
2	pH	2	Topographic index
3	Zn_*E*_	3	Enhanced vegetation index
4	Zn_DTPA_	4	Slope
5	SOC	5	Downscaled mean annual temperature
6	Zn_AR_		
7	eCEC		
8	Oxalates		
9	Zn_*K*d_		

*The subscripts AR, S, DTPA, *K*d and *E* denote the total (aqua regia extractable), soluble (calcium nitrate extractable), potentially available (DTPA extractable), the solid-solution distribution coefficient, and the isotopically exchangeable (isotopic dilution) fractions, respectively. SOC denotes soil organic carbon. eCEC denotes effective cation exchange capacity. Oxalates denotes the sum of oxalate-extractable Fe, Al and Mn oxides.

The isotopically exchangeable Zn (Zn_*E*_) and DTPA-extracted Zn (Zn_DTPA_) are capacity measures reflecting the reservoir of Zn in the soil available to re-supply that which has been removed from the soil solution^13^. The former variable was ranked first because DTPA is used at a small concentration (0.005 M) which may not be a fully efficacious extractant. The ‘total’ Zn extracted by aqua regia (Zn_AR_) was ranked lower because it may include soil Zn which is unavailable to plants. Soil organic carbon (SOC) was included after Zn_DTPA_ and ahead of Zn_AR_. This was based on evidence for larger concentrations of Zn in maize grain growing on soils with larger organic inputs^15^, potentially through effects on Zn availability and as a source of Zn. The effective cation exchange capacity (eCEC), and the sum of oxalate-extractable Fe, Mn and Al oxides were then included; these properties of the soil may influence the overall capacity to retain cations such as Zn. The solid ⇌ solution distribution coefficient (Zn_*K*d_), which is a measure of how strongly the soil adsorbs Zn, was included last.

The first-ranked environmental covariate was down-scaled precipitation, reflecting conditions for plant growth and which also influence soil climate and processes such as immobilisation. The topographic index is informative about the distribution of water in the landscape and so the local soil climate and water supply to the crop, but also relates to position in the soil catena moving from the erosion on interfluves to the accumulating positions at the topographical bottom. The remotely sensed vegetation index was then included, as a direct measure of the environment for plant growth as influenced by water supply and soil conditions. Slope was included as an additional variable reflecting landscape position. Downscaled mean annual temperature was included last as a potential proxy variable for altitude and associated environmental factors.

### Model-fitting

**Figure 1:**
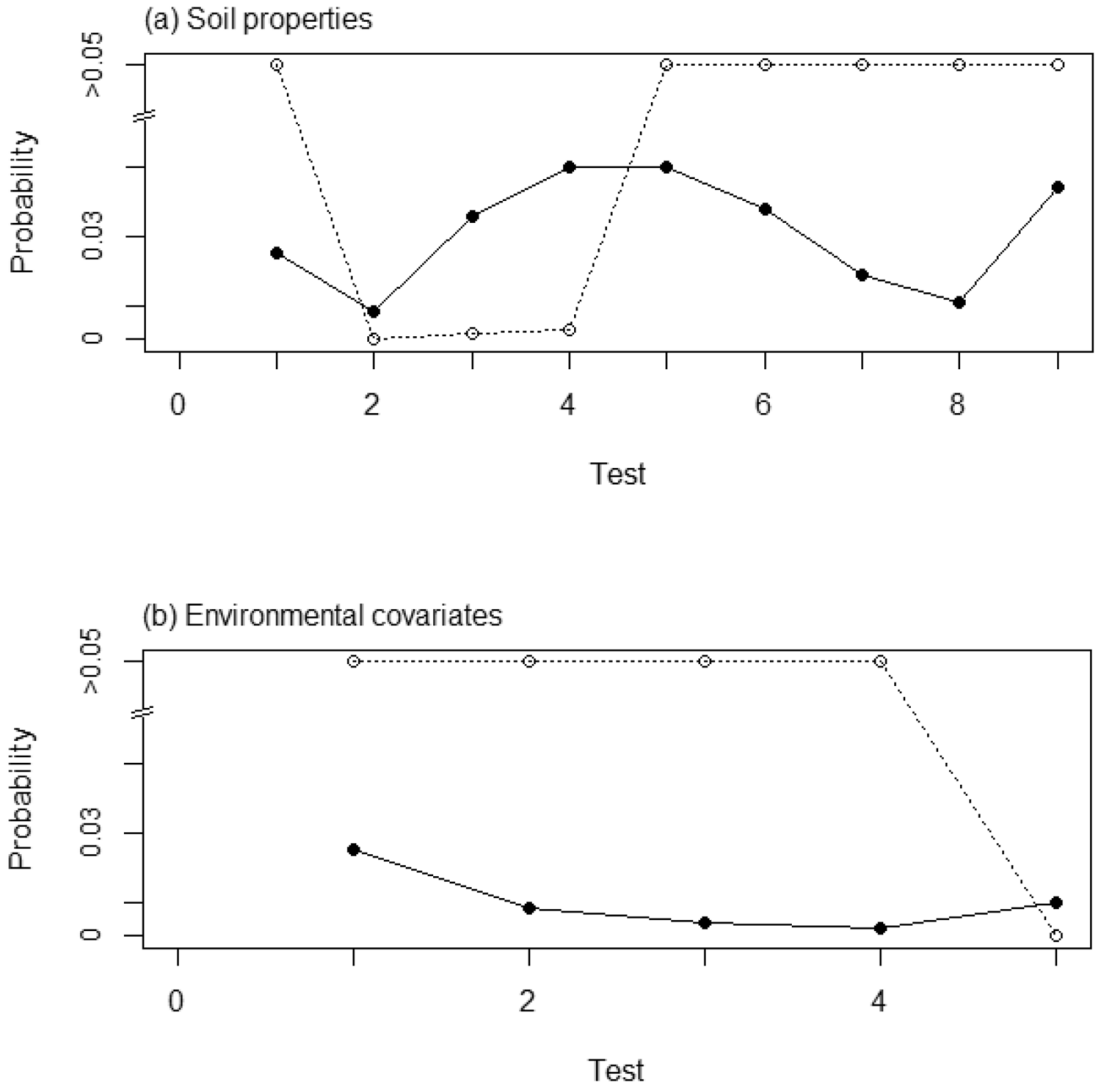
The *p*-values (open circles) for successive tests on predictors added to the model for grain Zn from (**a**) soil properties and (**b**) environmental covariates. Tests are on addition of variables in the order given in Table 1. The solid circles are the threshold for rejection of each null hypothesis under the FDR control.

**Figure 2:**
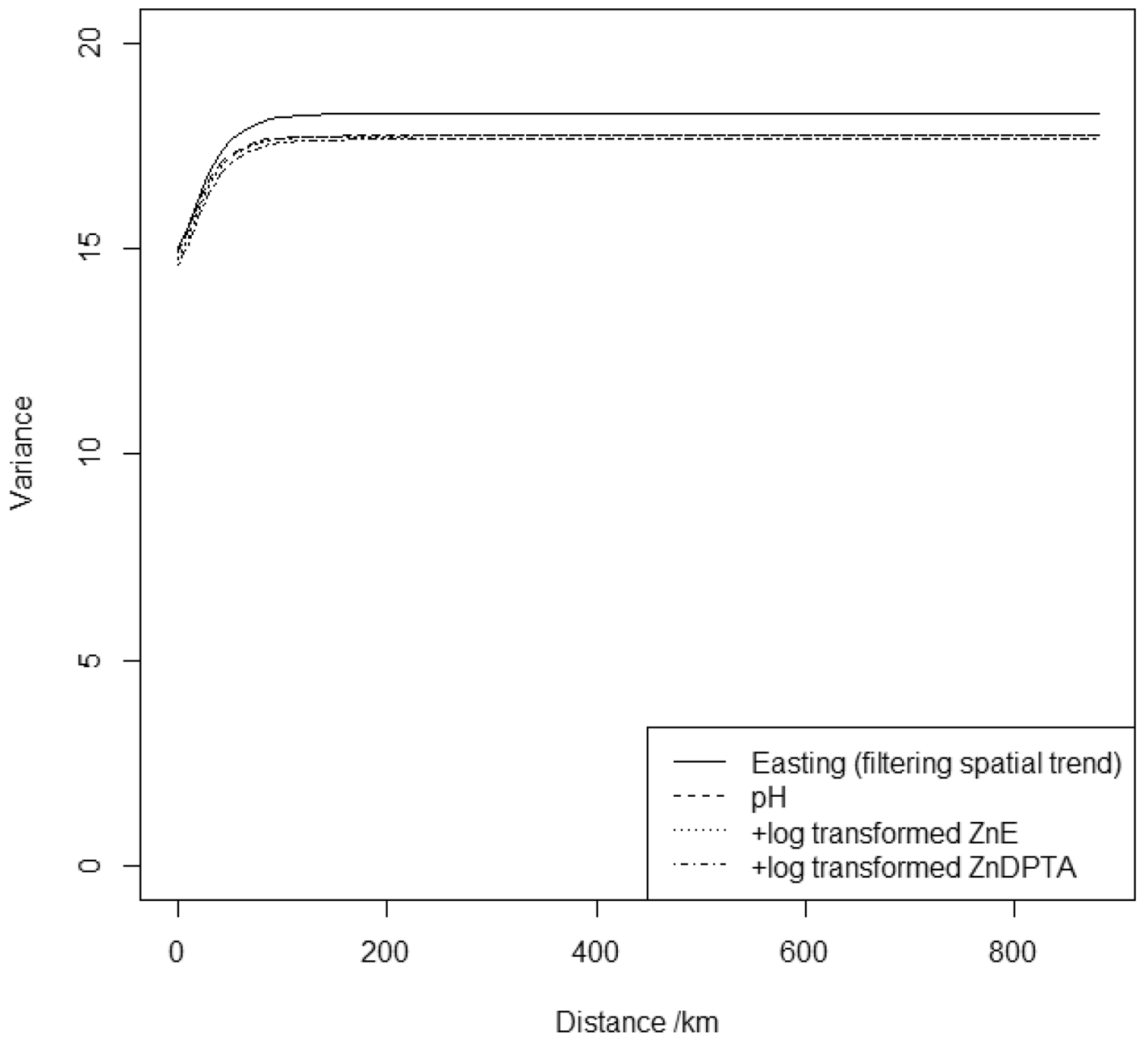
Variogram functions for the null model (eastings only) for maize grain zinc concentration, and for successive models with selected soil properties added as predictors.

The sequential fitting of models with predictors ordered as in Table 3 resulted in the retention of the 2nd- 3rd- and 4th-ranked soil properties, pH, Zn_*E*_ and Zn_DTPA_ as predictors for grain Zn concentration. The corresponding *p*-values and the corresponding thresholds under FDR control with α-investment are shown in Fig. 1a,b. Table 4 shows the estimated parameters for this model, relative to the null model with eastings as the only fixed effect. Figure 2 shows the variogram functions for the null model (eastings the only fixed effect) and for the models in which the selected soil predictors are added in succession. The variogram function summarises the variance parameters of the random effects in the LMM. The variogram is half the expected squared difference between two observations of the variable as a function of the distance between them. The variance of the iid random effect (the nugget variance) is a constant component of this value, and there is an additional contribution from the correlated random effect which depends on distance in a way determined by the f and k parameters. The sum of variances of the iid and correlated random effects is the upper bound of the variogram and gives the variance in the dependent variable not accounted for by the fixed effects. The reduction in this variance achieved by adding the soil properties is a small proportion (0.034) of the value of the variance for the model with eastings only, this is the quantity reported as the approximate adjusted R^2^ value for the model in Table 4 R^2^_adj_, equal to the difference of the sums of the two variance components for the null model and the model with predictors included expressed as a proportion of the sum of variance component of the null model. However, note that most of the variance in both models comes from the iid component, τ^2^. This is the so-called nugget variance, partially attributable to sources of variation spatially correlated over very short distances and also potential measurement errors (which are likely to be small). Most of the random variation in grain Zn is therefore fine-scale, representing factors varying over short distances and Fig. 2 shows that this quantity is unaffected by the inclusion of soil properties as predictors. A larger proportion of the spatially correlated variation seen in the variable, R^2^_adj,c_ (0.069), is accounted for by the inclusion of the soil properties as fixed effects, although much remains unexplained. The quantity R^2^_adj,c_ is the difference between the variance of the correlated random effects, σ^2^, for the null model and the model with predictors included, expressed as a proportion of the correlated variance for the null model. The effect of this small, though statistically significant, success of the model in accounting for variation in Zn grain concentration is seen in the somewhat smaller upper bound on the variograms for the models with the soil properties included (Fig. 2).

**Table 4:** Fitted models for soil properties and maize grain Zn concentration in Malawi.

Predictand		Predictor and coefficient					$R$^2^_adj_	$R$ ^2^_adj,c_	κ	τ^2^	σ^2^	ϕ
		*β_0_	β_1_	β_2_	β_3_	β_4_						
Maize Zn			Easting	pH	**Zn_*E*_	Zn_DTPA_						
	Null model	9.884	0.019						1.0	14.995	3.277	21.123
		8.420	0.018	0.4027	− 0.0736	0.6007	0.0342	0.069	1.0	14.596	3.051	20.867

*β_0_–β_4_, fixed effects coefficients β_0_ is a constant and β_*i*_ is the coefficient for the *i*th random effect; R^2^_adj_, the difference between the sum of the variances of the random effects for the null model and the proposed model expressed as a proportion of the sum for the null model; R^2^_adj,c_, the difference between the variance of the correlated random effect (σ^2^^2^, variance of the iid random effect (nugget variance); σ^2^, variance of the correlated random effect; ϕ, distance parameter of the Matérn correlation function.

**The subscripts *E* and DTPA denote the isotopically exchangeable (isotopic dilution), and potentially available (DTPA extractable) fraction, respectively.

Soil pH has a positive coefficient, indicating that, overall, the less acid soils have a larger concentration of Zn in grain. There is also a positive coefficient for Zn_DPTA_ and a smaller but negative one for Zn_*E*_. The mechanistic interpretation of these coefficients must be approached with caution because they partly reflect the mutual correlation among the fixed effect variables, and so are conditional on the other variables present in the model.

**Figure 3:**
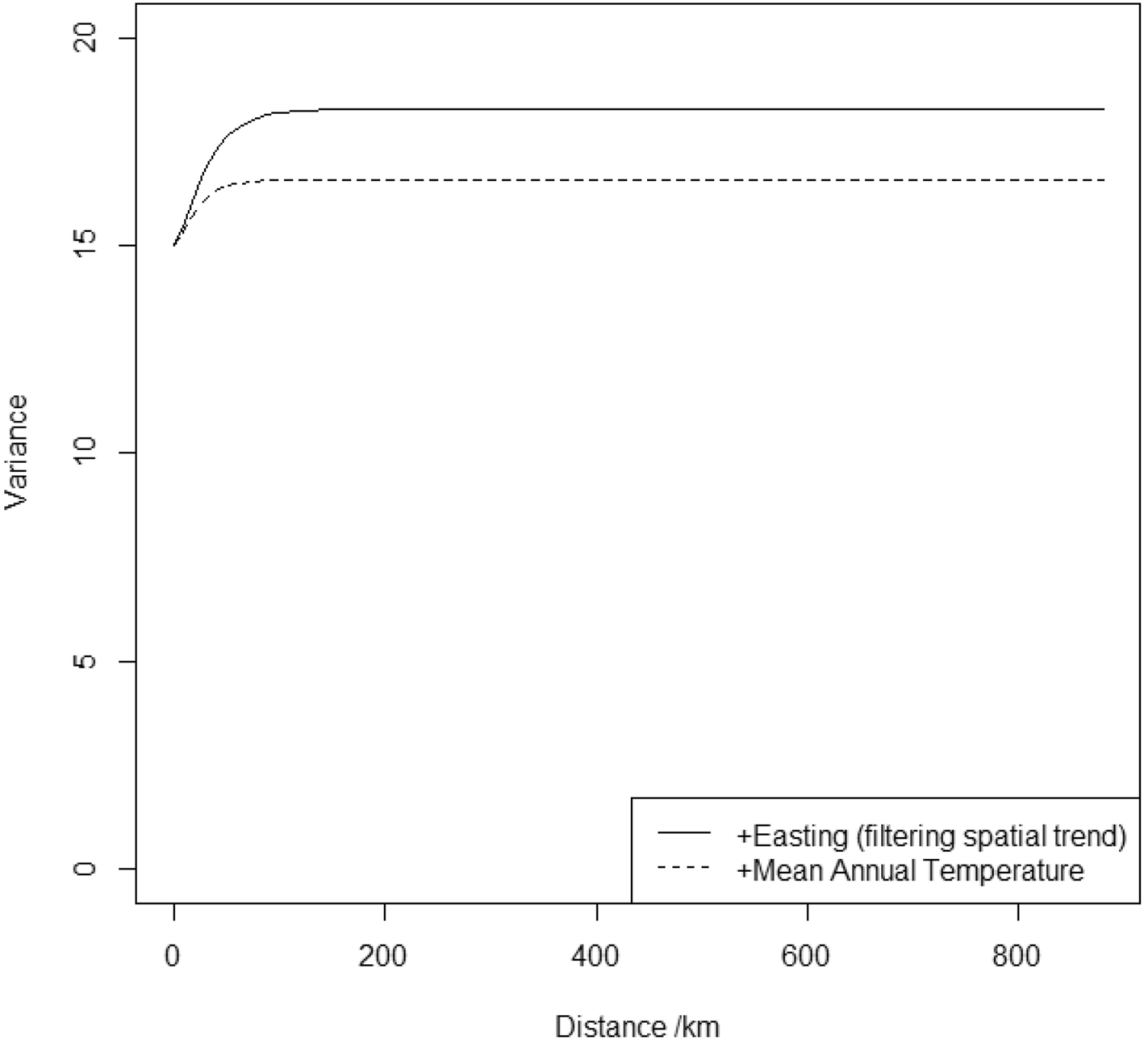
Variogram functions for the null model (eastings only) for maize grain zinc concentration, and for successive models with selected environmental covariates added as predictors.

The only environmental covariate selected by the FDR procedure is the last-ranked one, a positive coefficient for mean annual temperature. Table 5 shows the estimated parameters for this model, relative to the null model with eastings as the only fixed effect, and Fig. 3 shows the variogram functions. As with soil properties, the overall proportion of the random variation in grain Zn accounted for by the fixed effects is small (0.093), but again a much larger proportion of the spatially correlated variance is reduced by inclusion of the covariate (0.523).

**Table 5:** Fitted models for environmental covariates and maize grain Zn concentration in Malawi.

Predictand		Predictor and Coefficient			$R$^2^_adj_	$R$^2^_adj,c_	κ κ	τ^2^	σ^2^	ϕ
		*β_0_	β_1_	β_2_						
Maize Zn			Easting	Mean Annual Temperature						
	Null model	9.884	0.019				1.0	14.995	3.277	21.123
		− 0.222	0.010	0.073	0.093	0.523	1.0	15.008	1.564	15.592

*β_0_–β_4_, fixed effects coefficients β_0_ is a constant and β_*i*_ is the coefficient for the *i*th random effect; R^2^_adj_, the difference between the sum of the variances of the random effects for the null model and the proposed model expressed as a proportion of the sum for the null model; R^2^_adj,c_, the difference between the variance of the correlated random effect (σ^2^) for the null model and the proposed model expressed as a proportion of that variance for the null model; κ, smoothness parameter of the Matérn correlation function; τ^2^, variance of the iid random effect (nugget variance); σ^2^, variance of the correlated random effect; ϕ, distance parameter of the Matérn correlation function.

The summary statistics for the cross-validation errors are in Table 1 and their exploratory plots are in Figure S3. The assumption of normal errors appears plausible. The mean standardised squared prediction error is 1, but the median is 0.37. This is smaller than expected; the 95% confidence interval for the median under a valid model is [0.40, 0.51], and the kriging variances may be somewhat large, possibly due to outlying observations in the data, so inferences will be conservative in the sense that uncertainty is slightly overestimated.

### Spatial mapping

**Figure 4:**
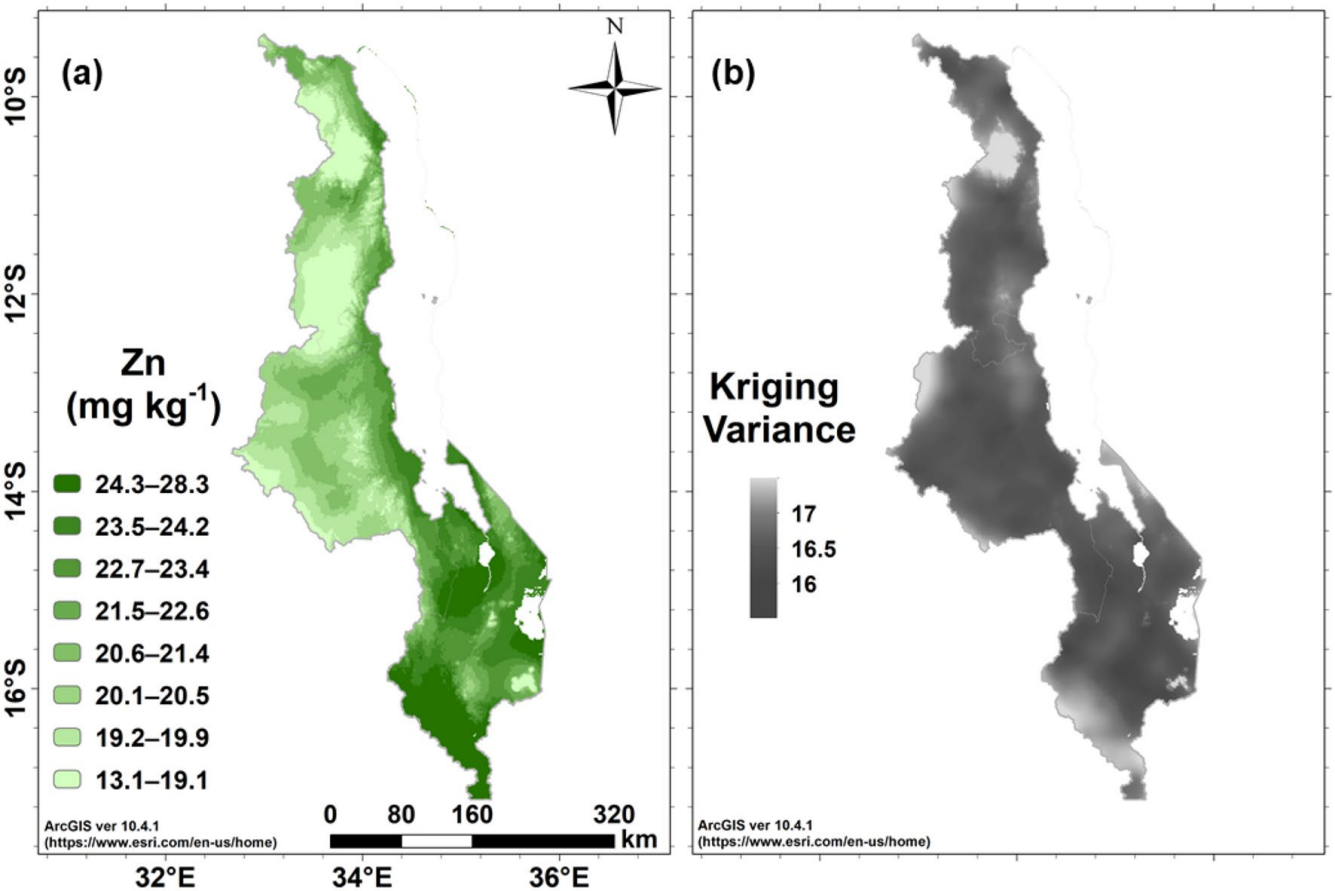
Grain Zn concentration in maize grain across Malawi. (**a**) Empirical Best Linear Unbiased Predictions, and (**b**) the prediction error variance (expected squared error) of the E-BLUP.

Spatial patterns of grain Zn concentration show larger values in the Shire valley in the south, and along the margins of Lake Malawi (Fig. 4a). These trends in grain Zn at national scale should be interpreted with caution over small regions where the covariate changes markedly. The prediction error variances in Fig. 4b show the variation in uncertainty over the country. For example, the concentrations are smaller around the Mulanje Massif in the south east of the country and the Nyika Plateau in the North. Whilst this may reflect the influence of the mean annual temperature covariate, the kriging variances are greater over the Mulanje Massif and the Nyika Plateau.

**Figure 5:**
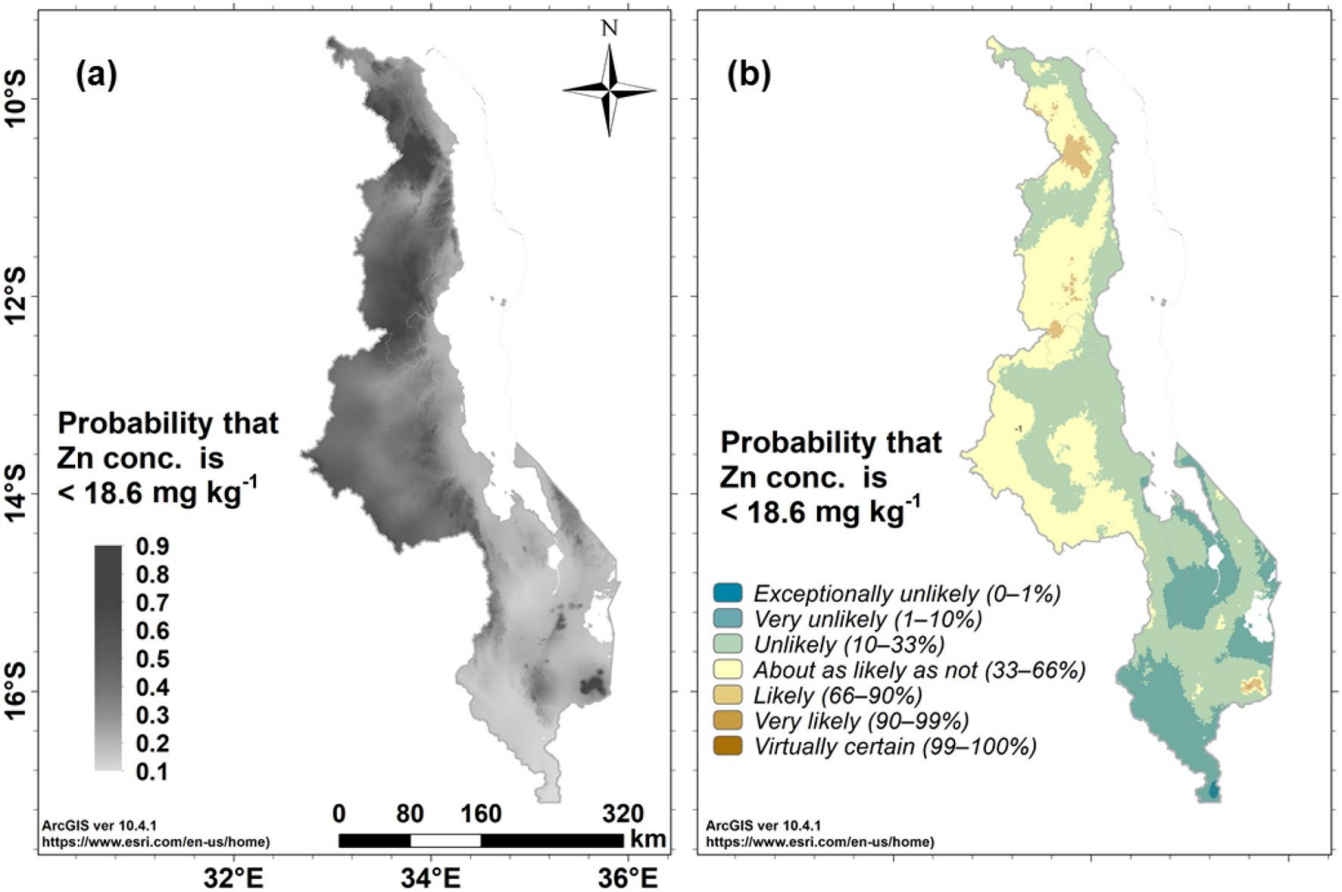
Probability that the concentration of Zn in maize grain across Malawi is < 18.6 mg kg^−1^ based on (**a**) numerical scale, (**b**) expressed according to ‘calibrated phrases’.

Figure 5a shows the probability that Zn grain concentration falls below a threshold of 18.6 mg kg^−1^. This threshold was defined as supplying a proportion of the Zn dietary Estimated Average Requirement (EAR) that is equivalent to the proportion of 50% dietary energy intake from maize in a typical diet. In Fig. 5b, these values are presented as ‘calibrated phrases’ to communicate probabilistic information about uncertain variables^6,16–18^. Over much of the central, northern and more western parts of the country the probability is in the interval 33–66%, interpreted as ‘about as likely as not’. In these regions further measurements would be needed before deciding whether interventions are needed, for example agronomic biofortification by fertiliser application^19^, because the dietary supply of Zn from the locally-grown staple crop is small.

## Discussion

Based on a median grain Zn concentration of 21.5 mg kg^−1^ and a reference daily maize intake of 343 g capita^−1^ day^−1^ from food balance sheets^20^, the typical dietary intake of Zn from maize alone in Malawi is 7.4 mg capita d^−1^. This intake represents 72% of an EAR of 10.3 mg capita d^−1^ for Zn in adult woman of 18–24 years. However, the spatial structure observed in grain Zn concentrations from the survey, and their range from 10–48 mg kg^−1^ indicates that the dietary intake of Zn from the locally-produced staple maize crop could vary markedly for an individual, 3.4–16.5 mg Zn capita d^−1^ from maize, or 34–160% of the EAR for Zn.. This observation highlights the importance of the effect of location on the likely prevalence of Zn deficiency among populations, especially where there is a reliance on a single dietary staple crop^10^. From a previous dietary recall survey^7^, median Zn intake per capita was previously estimated to be 8.5 mg d^−1^ and with a median intake per Adult Male Equivalent (AME) of 10.0 mg d^−1^. Lower intakes are likely in rural areas and among poorer households who have more limited access to more Zn-rich food sources such as meat, fish, and vegetables^7^. These populations are also less likely to benefit from current large-scale food fortification policies, as they are likely to consume a smaller proportion of fortified foods^21^. From food supply data at a national level, it was previously estimated that Malawi has a dietary Zn supply of < 14 mg capita^−1^ day^−1^, compared with a supply of ~ 20 mg capita^−1^ day^−1^ in many higher-income countries^22^.

Geographical differences in Zn intake have been reported previously in Malawi, based on (1) direct compositional analysis of dietary intakes in two different locations^5^, and (2) from national-scale dietary surveys linked to food composition data based on convenience sampling^7^. The current spatially representative survey of maize grain Zn concentration is consistent with data from both of those earlier studies. Siyame et al.^5^ reported a median Zn intake among adult women of 4.8 (1st and 3rd quartiles 3.5, 6.4; n = 55) and 6.4 (1st and 3rd quartiles, 3.5, 6.4; n = 58) mg capita^−1^ d^−1^ in Zombwe and Mikalango Extension Planning Areas (EPAs), respectively. Zombwe EPA is in the Northern region (within a light green area in Fig. 4, representing low grain Zn concentrations), compared with Mikalango EPA which is in the Southern region (a dark green area in Fig. 4, representing high grain Zn concentrations). Siyame et al. noted that these differences corresponded with a reported ~ 30% greater grain Zn concentration in maize growing on Vertisol soil types of higher pH in Mikalango EPA, than maize growing on the more acidic soils typical of Zombwe EPA, as reported earlier by Chilimba et al.^8^. Joy et al.^7^ also used data from Chilimba et al. together with new food composition data obtained by convenience sampling^23^. Among 179 EPAs across the country, median Zn supply per AME ranged from 4.4 mg d^−1^ in Kalumba EPA (n = 16 households) to 15.8 mg d^−1^ in Masambanjati EPA (n = 32). Again, these contrasts are consistent with the current study, whereby Kalumba EPA is in the Central region, and falls within an area shaded lighter green (maize grain Zn concentration of 13.1–21.4 mg kg^−1^), and Masambanjati EPA which is in the Southern region, and falls within an area shaded darker green (maize grain Zn concentration of 21.5–24.2 mg kg^−1^).

The predictive value of soil factors for maize grain Zn concentration was significant, albeit the three factors reported here (pH, isotopically-exchangeable Zn, DTPA-extractable Zn) explained only a small proportion of the random spatial effects (adjusted *R*^2^ = 0.034) within the overall model, once the fixed spatial trend effect had been accounted for. A positive relationship between soil pH and maize grain Zn concentration is consistent with contrasts reported between soil types^5,8,23^, and with trends reported for maize grain Zn concentration in Ethiopia and Malawi^10^. Furthermore, increasing soil pH has been shown to increase the pool of isotopically exchangeable Zn in soils, and therefore its potential plant availability^13^. In this study, the isotopically exchangeable Zn fraction had significant predictive power for maize grain Zn concentration, as did DTPA-extractable Zn (a more conventional measure of plant Zn availability in soils), even after controlling for the effects of soil pH using FDR adjustments.

Relationships between SOC and grain Zn concentration were not observed in this current analysis. In a previous study using these data, in which soil pH and SOC were the only two soil factors included in the analysis, a significant but weak positive correlation was observed between SOC and maize grain Zn concentration in both Ethiopia and Malawi^13^. That previous report was consistent with survey-based observations that the preferential use of locally sourced organic materials by smallholder farmers can improve grain Zn concentrations in maize (in Zimbabwe^15^) and wheat (Ethiopia^24^) cropping systems. A lack of association between SOC and maize grain Zn concentration in this analysis should be treated with caution. Whilst the SOC effect was > 0.05, it is possible that the predictors included previously in the analysis are correlated with SOC, and so act as a proxy. For example, DTPA-extractable Zn and SOC were shown to be correlated in the studies of both Manzeke et al.^15^ and Mossa et al.^13^.

The positive relationship between mean annual temperature and grain Zn concentration had the largest predictive power of all the soil properties and environmental covariates tested in this study. However, this factor still only accounted for a relatively small proportion of the variation in grain Zn concentration due to random spatial effects (adjusted *R*^2^ = 0.093), once the fixed spatial trend effect had been accounted for within the overall model. The reason why a greater mean annual temperature leads to increased maize grain Zn concentration may be due to smaller grains and greater transpiration occurring under warmer conditions. This observation could be used to support the design of future surveys, which might be able to incorporate further information on temperature and other climate-related and spatially correlated landscape factors.

Strengths of the current study include the use of a formal hypothesis testing framework and FDR control based on expert ranking of potential soil properties and environmental covariates which influence maize grain Zn concentration. Such a robust statistical approach is well-suited to this type of survey in which the sampling frame was conditioned to maximise the spatial coverage of a geographical area. Using this approach, we have identified soil properties (pH, isotopically exchangeable Zn, DTPA-extractable Zn) and an environmental covariate (mean annual temperature) that explain some of the spatially correlated variation in maize grain Zn concentration. This study was not designed to quantify the effects of how crop variety (Genotype, G) or farmer practices (Management, M) will influence grain Zn concentrations. Genotypic variation in maize grain Zn concentration is already being used to breed biofortified varieties of maize, for example, the grain Zn concentration of new hybrid maize varieties released in Guatemala (ICTA HB–18, ICTA B-15) and Colombia (BIO-MZN01) are reported^25^ to contain 15% and 36%, respectively in the two countries, above target levels of ~ 30 mg kg^–1^. Multiple agronomic factors will affect yield, yield components, and grain micronutrient concentrations across multiple scales. These include the use of both standard NPK- and Zn-containing fertilisers and the use of organic soil amendments, which can increase maize grain Zn concentrations^15,19,26^. Future surveys could be designed to determine the contribution of G and M factors, together with complex G × E × M interactions, to gain a greater more understanding of the factors which influence variation in grain Zn concentration, and to design practical interventions to increase the dietary supply of Zn through crops.

## Materials and methods

The field sampling and analyses of grain, soil pH, and SOC was described previously by Gashu et al.^10^, with further details provided here.

### Sampling design

The sampling domain was defined as all raster cells in the European Space Agency Climate Change Initiative data within Malawi^27^ for which ‘cropland’ was used in the land use description. The primary objective of this sampling was to support spatial prediction for mapping of the variables measured. Such a sampling design requires good spatial coverage, to minimise the distance between any location where a prediction is made and the nearest sample to support that prediction^28^. It also requires an additional subset of close pairs of points to support the statistical modelling required as a basis for spatial prediction^29^. We formed an initial ‘spatial coverage’ target sample set of 1710 locations, including 820 fixed points from the 2015/16 Demographic and Health Survey of Malawi, 890 additional locations, plus a further 190 close-pair sample locations^10^.

### Field sampling

Sampling was undertaken by trained teams as described in Gashu et al.^10^. From a total of 1812 sites where grain and soil samples were taken, 1790 had location accuracy of ≤ 9 m. A further 16 sites had location accuracy of 10–17 m, whilst six locations had positional uncertainties of 2,900–5,000 m most likely due to either poor satellite signal or enumerators not giving the devices enough time to establish the location. These latter six locations, along with 204 locations from which crops other than maize were sampled (sorghum, rice, pearl millet, finger millet) were excluded from the analyses reported in this paper, giving a sample size of 1602 locations.

### Grain and soil analyses

Grain samples were prepared and analysed using methods described in Gashu et al.^10^. Briefly, Zn concentration in grain determined using inductively coupled plasma mass spectrometry (ICP-MS; iCAPQ, Thermo Fisher Scientific, Bremen, Germany) following acid digestion with 6 mL of 70% Trace Analysis Grade (TAG) HNO_3_ in a Multiwave microwave. Soil parameters were determined using methods described in Mossa et al.^13^ and Gashu et al.^14^: soil pH_(water)_ in a soil:water suspension ratio of 1:2.5; total nitrogen (N); total carbon (C, dry combustion); soil organic C (SOC) based on the difference between total C and measured inorganic C; effective cation exchange capacity (eCEC) and exchangeable cations using hexaminecobalt trichloride solution; amorphous oxides (AlOx, FeOx, MnOx) using ammonium oxalate extraction; Olsen P; DTPA-extractable Zn; isotopically exchangeable Zn (Zn_*E*_). Two data points were excluded, one with a grain Zn concentration less than the limit of detection and one with a missing Zn_*K*d_ value, giving a final sample size of 1600.

### Extraction of environmental covariates

A set of environmental covariates, possibly correlated to Zn status of grain through effects on crop growth and soil conditions, were identified for consideration. We obtained the MERIT Digital Elevation Model^30^, and the associated values for surface slope and the topographic index, which measures the tendency of water to accumulate at a location in so far as this is determined by overland flow. We also obtained the downscaled values for mean annual temperature and precipitation in the CHELSA data set^31^. Finally, we obtained the Enhanced Vegetation Index (EVI) based on data from the MODIS remote sensor satellite^32^. Specifically, we used the average value of the 250-m EVI product (MOD13Q1) over the period 2000–2016.

### Data analyses

#### Linear mixed model (LMM)

There are two overall objectives. The first is to identify relationships between soil properties and the concentration of Zn in maize grain. The second is to model the spatial variation of Zn concentration in maize grain at national scale to support mapping of this variable, exploiting any relationships with the environmental covariates for which there is evidence. A Linear Mixed Model (LMM) framework was used in which the variable is modelled as a combination of fixed effects (linear functions of soil properties or environmental covariates), a correlated random effect, and an independent and identically distributed (iid) random error (nugget effect) which incorporates variation due both to measurement error and factors that vary over short distances relative to the spacing of sample points. A LMM is necessary for modelling to support spatial prediction, and for testing hypotheses about relationships between grain Zn concentration and soil properties, because the sample sites were not selected by a probability sampling design that would enable them to be treated as independent and modelled using other methods, such as ordinary least squares regression^28,33^. Here, the random effect was a Gaussian random field with a spatial covariance structure^33,34^. This spatial LMM provides a basis for interpolation of the variable of interest, whereby the prediction error variance (expected squared error) is minimised conditional on the LMM. This prediction is called the best linear unbiased predictor (BLUP), and the BLUP based on a fitted LMM to data is called the empirical BLUP (E-BLUP). For a spatial LMM, in which the only fixed effect is an unknown mean, the E-BLUP is equivalent to the ordinary kriging prediction^35^, if spatial covariates are incorporated in the fixed effects then the E-BLUP is equivalent to the kriging prediction with an external drift.

### Statistical inference and false discovery rate (FDR) control with α-investment

In a LMM framework, the evidence that a fixed effect coefficient is significantly different from zero can be tested by calculating the Wald statistic^33^, and the evidence that adding fixed effects to a simpler model achieves a significant improvement by computing the log-ratio statistic:

1 $$L = 2\left( {\ell _{1} - \ell _{0} } \right)$$

where ${\ell _{1}}$ and ${\ell _{0}}$ denote, respectively the maximised log-likelihoods from fitting the model with the additional fixed effects, and the simpler model without them. Under the null hypothesis, where the additional fixed effects are not related to the dependent variable, this statistic is asymptotically distributed as chi-square with degrees of freedom equal to the number of additional fixed effects.

We used this approach to hypothesis testing for the identification of independent variables (covariates) to include as fixed effects in models for target properties of interest. However, a step-wise procedure in which candidate predictors are considered and accepted or rejected on the basis of a hypothesis test is not a robust approach to variable selection^36,37^. A statistically consistent approach to variable selection is to treat it as a problem in multiple hypothesis testing^38^. That is to say, to recognise that the sequence of tests in step-wise variable selection do not constitute a set of tests of single hypotheses, each of interest in themselves, but rather that the interest is in which subset of candidate covariates appears to be related to the target variable^39^. The probability that a set of tests, all of which are of null hypotheses, result in incorrect rejections may be substantially larger than the threshold at which individual tests are conducted. One approach to multiple hypothesis testing is to control the false discovery rate (FDR) over a set of tests^40^. The FDR is the proportion of false rejections of null hypotheses out of those rejected. Various methods have been proposed to control FDR over a set of multiple tests^41^. A FDR control does reduce the power to detect real effects among a set of predictors. One way to reduce this loss of power, while maintaining FDR control, is by the method of α-investment^42^. This can be applied to a sequence of tests, and the power to detect real effects is increased when those null hypotheses most likely to be false (on the basis of a priori expectation, not exploratory analysis of data on the dependent variable) are tested at the beginning of the sequence. This is because the threshold against which the *p*-value for a test in the sequence is examined can be varied according to a quantity called the α-wealth which is depleted when null hypotheses are accepted and increased when they are rejected, while still controlling FDR.

Here, we used FDR control with α-investment^43^. Models were fitted sequentially, starting with a ‘null model’ with the only fixed effect a spatial trend identified in exploratory analysis of the data. This model was used, rather than a model with a constant mean as the only fixed effect, because the latter model would violate assumptions of second-order stationarity when a spatial trend is pronounced^35^. The null model was fitted by maximum likelihood (ML). The first predictor was then included as a fixed effect and the model refitted. The log-likelihood ratio statistic (Eq. 1) was then computed. If the *p*-value for this test exceeded 0.05 then the predictor was dropped, otherwise it was provisionally retained, and the next predictor was considered. Once all the predictors had been considered the *p*-values for each were compared to thresholds according to the α-wealth controlling FDR (here 0.05). The predictors for which the *p*-values fell below the α-wealth (FDR threshold) were then definitively retained, and the final model refitted this time by residual maximum likelihood (REML).

To apply this method, we required separate rankings of the soil properties and the environmental covariates as potential predictors of the Zn concentration in maize grain. These rankings were based on a priori expectations, based on understanding of the processes involved, and not from exploration of relationships between the available predictor variables and the target variables of interest. However, once a predictor is included in the ranking, a second predictor strongly correlated with it is unlikely to include much additional information about the target variable, and so is placed lower in the ranking that it would be based on process considerations alone. Whilst a poorly-informed ranking, which fails to reflect the relative importance of a predictor, can reduce our power to detect real effects as significant, the FDR is always controlled at the specified rate. An approach to variable selection based on FDR control with α-investment is therefore conservative, in the sense that FDR control reduces the risk of fitting an unduly complex model as a result of multiple hypothesis testing, and that an effective ranking simply increases the power of the procedure to detect those predictor variables for which there is evidence for a relationship with the target variable.

Rankings were provided by a panel meeting of soil and plant scientists. Before the meeting, the two lists of potential predictors available (soil properties and environmental covariates), and information on the correlations among the predictors within each group (Figure S4), were circulated among the panel members. Panel members were asked to share their views on the processes by which these might determine the concentration of Zn in grain. At the meeting, the principles of the approach to FDR control, and key points outlined in the previous two paragraphs were presented. The two sets of predictors were then ranked through a discussion, facilitated by statisticians with experience in group elicitation, who ensured that the discussion was not dominated by a few voices and that statistical concepts were understood correctly (e.g. how correlations between predictors might influence the process). Rankings of the two groups of predictors were obtained by a consensus.

### Exploratory data analysis and model-fitting

Summary statistics of the predictor variables were first examined along with the octile skewness coefficient^44^, which is robust to the effect of outlying observations. Whereas variables with (conventional) skewness in excess of 1.0 typically require transformation^35^, the equivalent guide value of the octile skewness is 0.2. Those with a pronounced octile skew coefficient were transformed to natural logarithms before they were used. The data on Zn concentration in maize grain were examined using the plot.geodata function from the geoR library^45^. This allows to examine the data for evidence of pronounced spatial trends by examining plots of the variable against the northings and eastings, and spatial plots of the data, coded with colour symbols to indicate the quartiles of the data set to which they belonged. ‘Saturated’ exploratory models for grain Zn concentration with (1) all soil properties as fixed effects, along with a trend in the eastings identified from the spatial plots, and (2) all environmental covariates and easting as fixed effects were then fitted, and the summary statistics and histograms of the residuals were examined to decide whether grain Zn concentration required transformation for an assumption of normality to be plausible in either or both cases. These exploratory models were fitted by ordinary least squares.

The LMMs were fitted using the likfit function in the geoR package for the R platform^45,46^. This method finds estimates of the parameters of the random effects by ML or REML. The former method is appropriate when two LMM with different fixed effects structures are to be compared. The latter is most appropriate when forming a model for prediction because bias in the estimated random effects is reduced when the fixed effects are more complex than a constant mean. The model-fitting procedure for LMMs is described previously^33,45^. Briefly, the estimated parameters for the random effects comprise the variance of the iid error (τ^2^), the variance of the spatially correlated random component (σ^2^), a distance parameter which quantifies the spatial scale over which the correlated random effect shows spatial dependence (ϕ), and a parameter which describes the smoothness of the spatial process (κ). This latter parameter can be challenging to estimate, so we used a profiling method^45^ in which the parameter is fixed at each of a set of discrete values and the others are fitted by ML. The value of κ for which the likelihood was largest was selected. This was done for the null model, and the selected value of κ was then used for all others.

The LMM with environmental covariates, to be used for spatial mapping, was examined by a cross-validation method in which each observation was extracted from the data set in turn and the E-BLUP and its prediction error variance computed from the remaining data. The summary statistics and exploratory plots of the errors were examined to evaluate the plausibility of assumptions of normal errors. The standardised squared prediction errors, the square of the difference between the observed value and the E-BLUP divided by the prediction error variance, were calculated and their mean and median values computed. We expected the mean value to be close to 1 and the median to be close to 0.455 for a valid model^47^.

### Spatial prediction

Once a LMM was fitted with selected predictors from among the environmental covariates, the E-BLUP was computed for each raster cell at which the selected covariates were recorded^33^. The prediction can be thought of as a combination of a ‘regression-type’ component, based on the selected covariate(s), and a ‘kriging-type’ prediction from the random effects. The prediction minimises the expected value of the prediction error variance, a quantity which is also calculated, and which quantifies the uncertainty of the prediction. The kriging variance along with the spatial predictions of Zn concentration were then presented as a map using ArcGIS (version 10.4.1; Environmental Systems Research Institute, ESRI, https://www.esri.com/en-us/home).

Because the uncertainty of E-BLUP predictions can be quantified in this way, one may also examine the uncertainties attached to interpretations of the map of grain Zn concentration. For example, the expected average requirement of Zn on a population-weighted basis^22^ is ~ 10.3 mg capita^−1^ day^−1^. Given a reference daily maize intake^20^ of 342.8 g capita^−1^ day^−1^, and an energy density for maize of 3.79 kcal g^−1^, the daily energy intake from maize is 342.8 × 3.79 kcal = 1,299.2 kcal, which is 61.9% of the average daily energy requirement (ADER) in Malawi^48^. Therefore, if maize is to provide the same proportion of the EAR of Zn, as it does of the ADER, then the concentration of maize in grain must equal 18.6 mg kg^−1^. A policy maker could therefore examine the map of grain Zn concentration provided here and identify where the concentration is below this threshold. However, these predictions are uncertain. If one assumes normal prediction errors, the probability that grain Zn falls below the threshold at a location can be computed from the normal distribution function, with mean equal to the E-BLUP at that location and variance equal to the E-BLUP prediction error variance. These probabilities were also presented as a map using ArcGIS (version 10.4.1; ESRI).

### Statement on grain samples

All the methods were carried out in accordance with relevant guidelines and regulations, including ethical approvals (as the samples were taken with the informed consent of the famers), permissions for sampling from relevant district and local authorities. Full details are in Gashu et al.^10^. Transfers of material between laboratories complied with national and institutional regulations^49^.

## Supplementary Information

Supplementary Information 1.

Supplementary Information 2.

Supplementary Information 3.

Supplementary Information 4.

## Data Availability

All the data are freely available from the corresponding author and available on line at https:/github.com/rmlark/GeoNutrition.
