# Supplementary material for: Soil and landscape factors influence geospatial variation in maize grain zinc concentration in Malawi
Source: Sci Rep. 2022 May 14;12:7986. doi: 10.1038/s41598-022-12014-w (PMC9107474; doi:10.1038/s41598-022-12014-w)

Figure S1. Histogram with boxplot and QQ plot for the residuals from an exploratory fit of the saturated model (soil properties) for concentration of Zn in grain

a

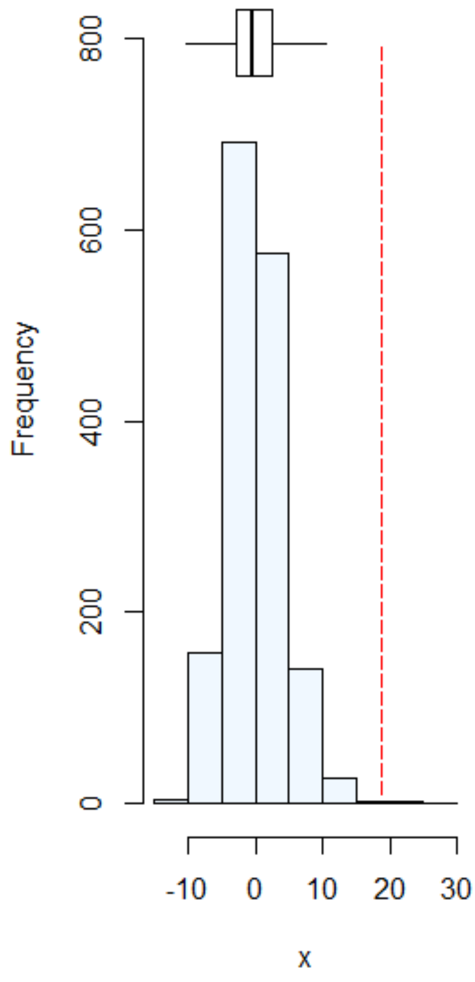

b

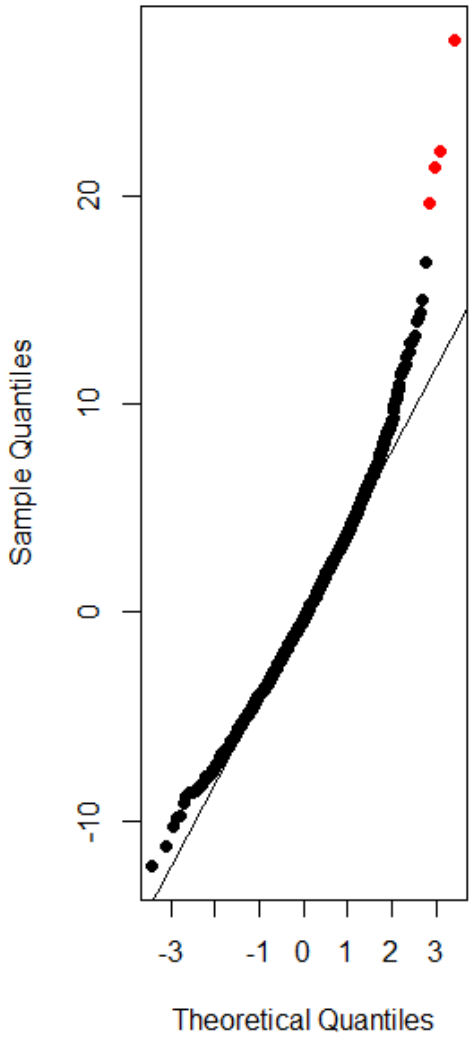

Supplement: Supplementary file 1 — Supplementary Information 1. [file 41598_2022_12014_MOESM1_ESM.pdf]
