# Supplementary material for: Soil and landscape factors influence geospatial variation in maize grain zinc concentration in Malawi
Source: Sci Rep. 2022 May 14;12:7986. doi: 10.1038/s41598-022-12014-w (PMC9107474; doi:10.1038/s41598-022-12014-w)

Figure S2. Histogram with boxplot and QQ plot for the residuals from an exploratory fit of the saturated model (environmental covariates) for concentration of Zn in grain

a **b**

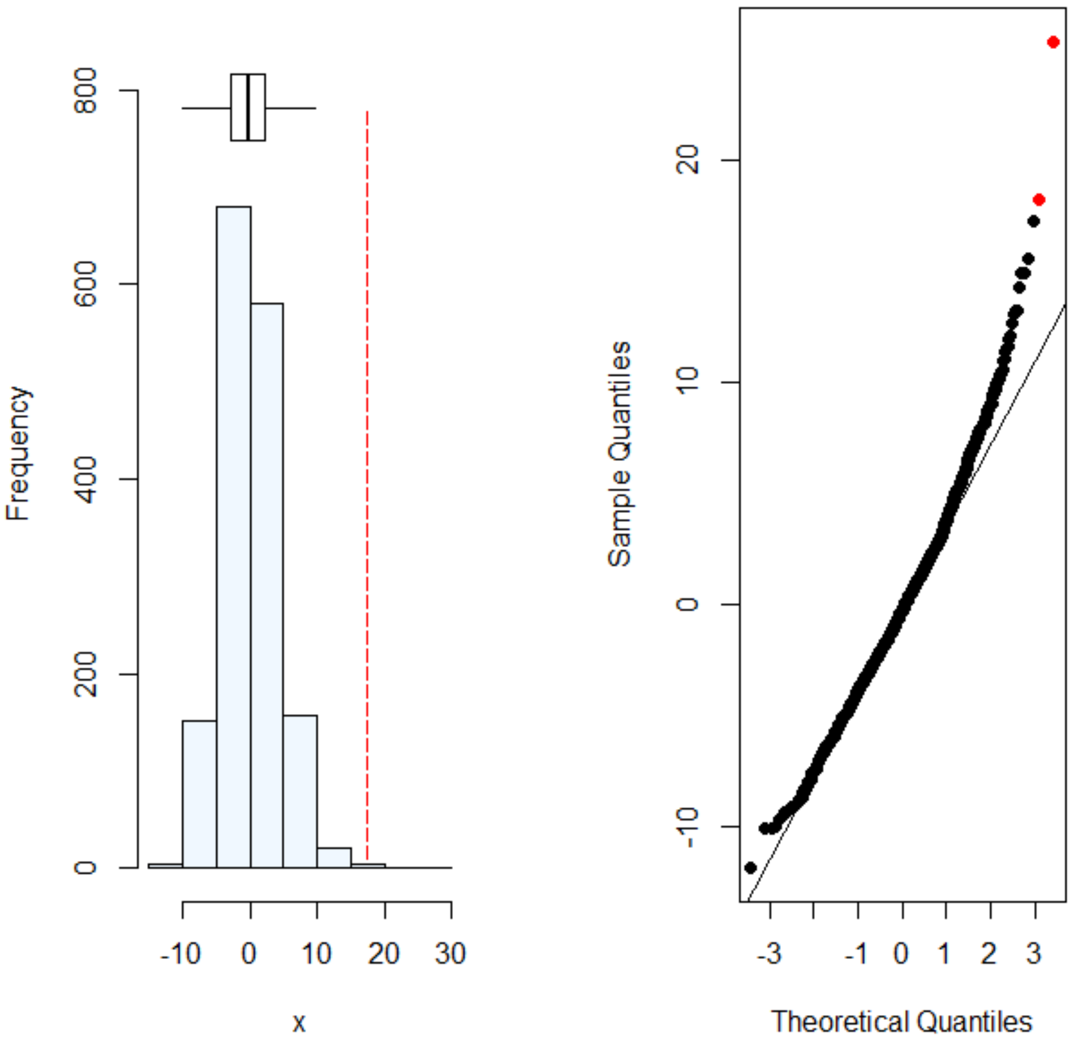

Supplement: Supplementary file 2 — Supplementary Information 2. [file 41598_2022_12014_MOESM2_ESM.pdf]
