# Supplementary figures and images for: Soil and landscape factors influence geospatial variation in maize grain zinc concentration in Malawi
Source: Sci Rep. 2022 May 14;12:7986. doi: 10.1038/s41598-022-12014-w (PMC9107474; doi:10.1038/s41598-022-12014-w)

**Figure S4. Relationships between soil properties and grain Zn concentration.**

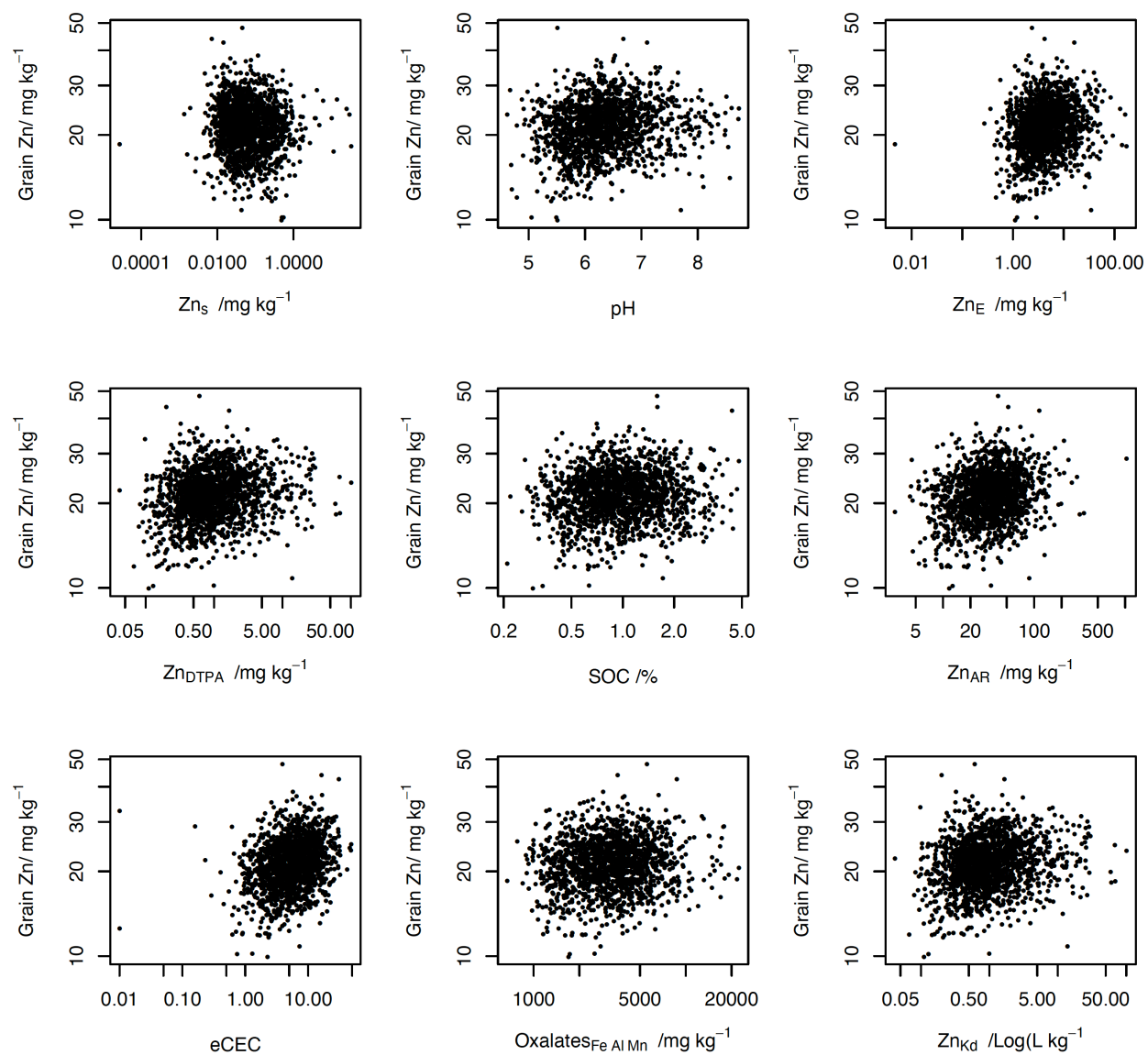

Supplement: Supplementary file 4 — Supplementary Information 4. [file 41598_2022_12014_MOESM4_ESM.pdf]
